# Supplementary material for: Structural basis of liprin-α-promoted LAR-RPTP clustering for modulation of phosphatase activity
Source: Nat Commun. 2020 Jan 10;11:169. doi: 10.1038/s41467-019-13949-x (PMC6954185; doi:10.1038/s41467-019-13949-x)
Supplement: Supplementary file 1 — Supplementary Information [file 41467_2019_13949_MOESM1_ESM.pdf]

Supplementary Information

Structural Basis of Liprin-α-promoted LAR-RPTP Clustering for Modulation of Phosphatase Activity

Xingqiao Xie et al.

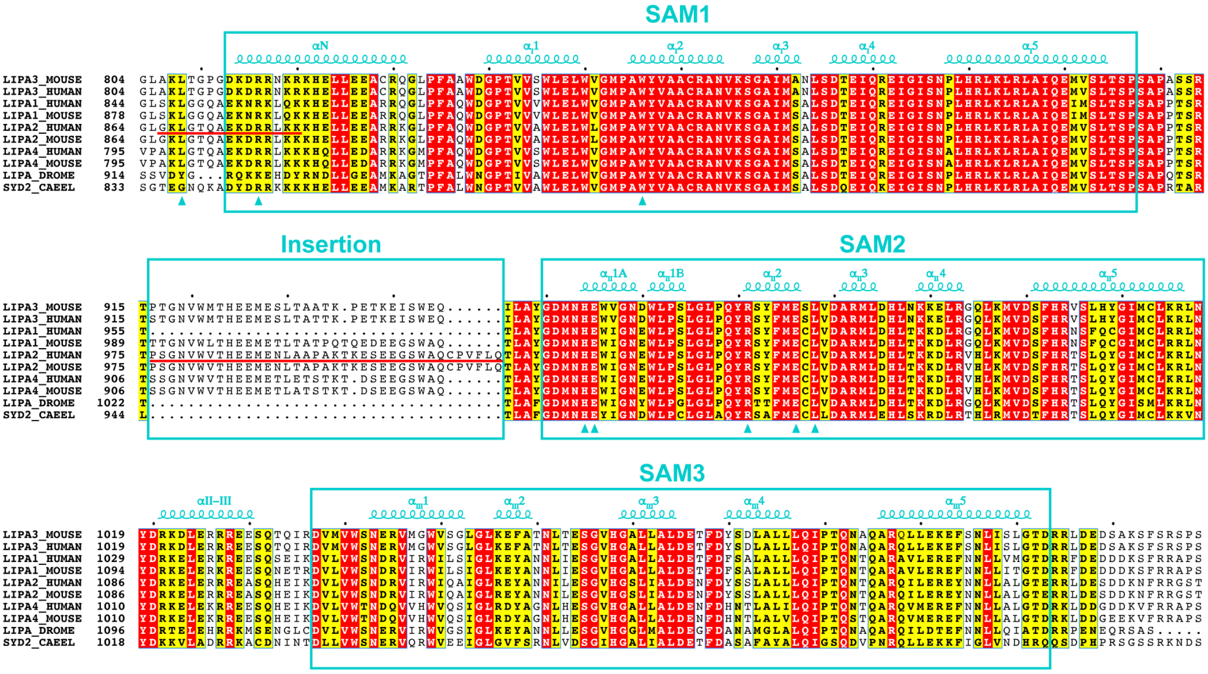

Supplementary Figure 1. Sequence alignment of the liprin-α family members from different species. “DROME” and “CAEEL” indicate *Drosophila melanogaster* and *C.elegans*, respectively. Residues involved in the liprin-α_SAM123/LAR_D1D2 interaction were marked with green triangles. The secondary structural elements were indicated above the alignment. The deletion regions in SAM123ΔN or SAM123ΔInsertion were highlighted by red lines.

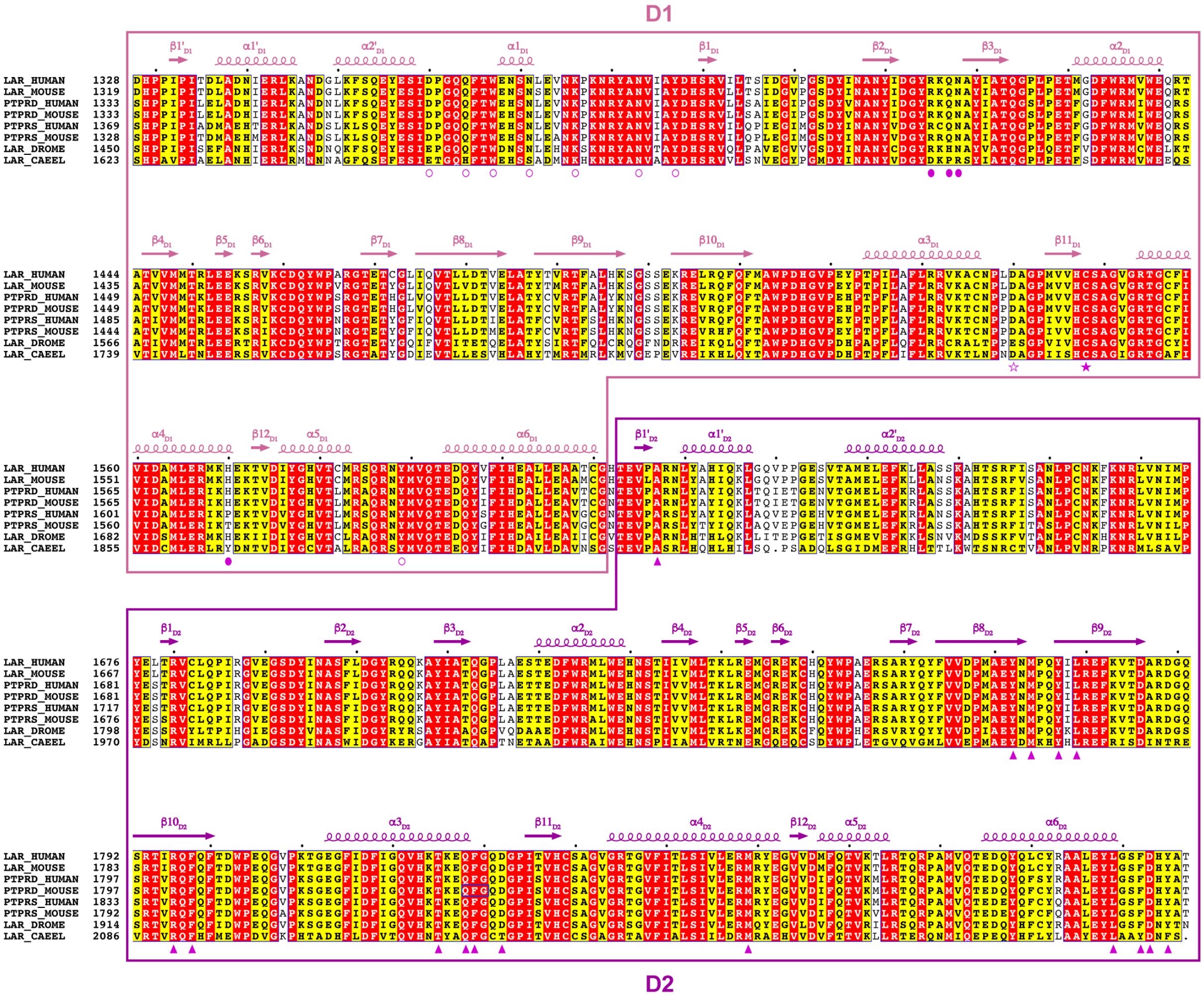

Supplementary Figure 2. Sequence alignment analyses of LAR from different species. Residues involved in the liprin-α_SAM123/LAR_D1D2 interaction were marked with purple triangles. Residues involved in the D1/D1 packing interface were marked with solid and open circles, respectively. In the D1 domain, the catalytic residue and the residue involved in blocking the substrate-binding site shown in Fig. 5b are labeled with a solid star and an open star, respectively. The secondary structural elements are indicated above the alignment.

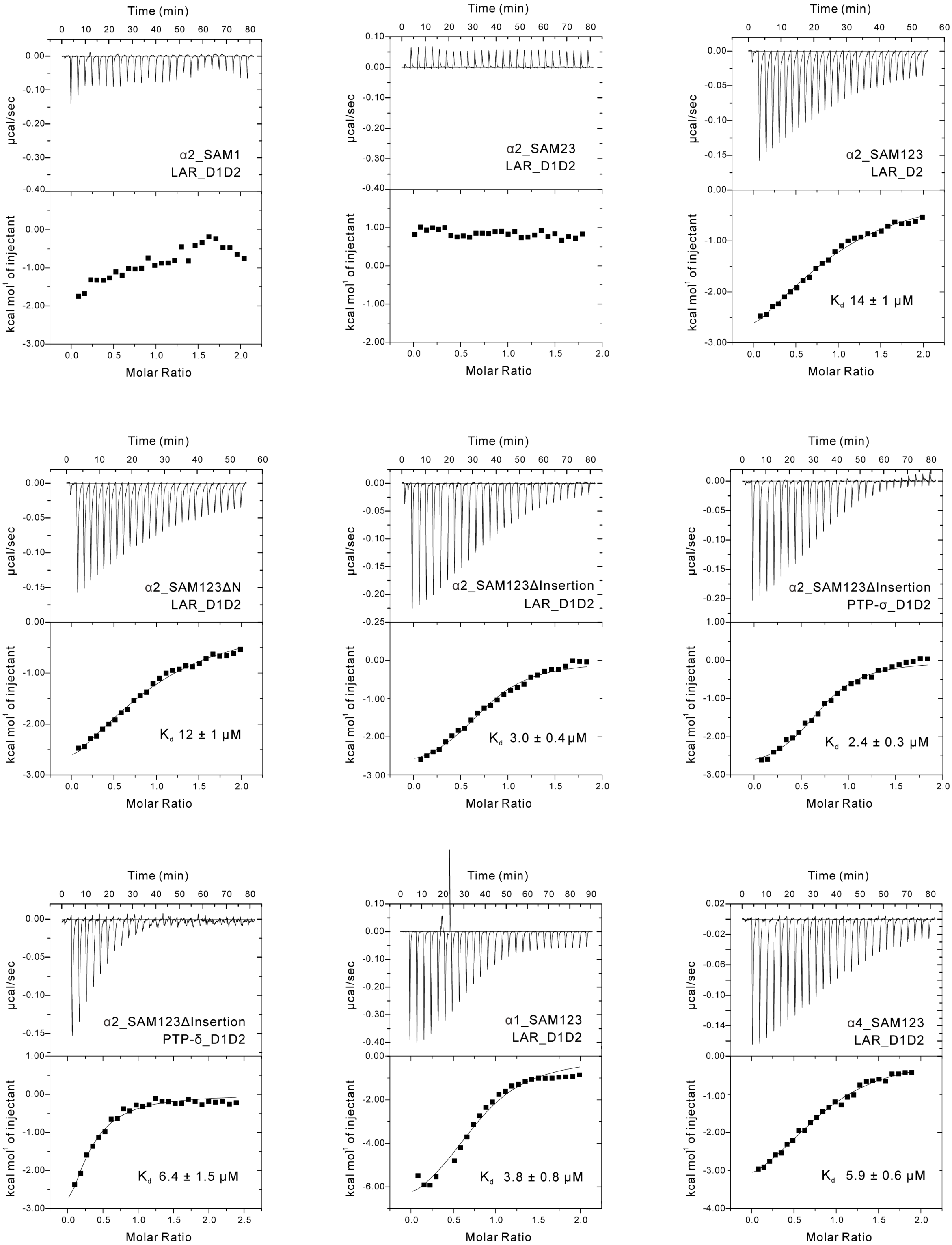

Supplementary Figure 3. ITC titration curves of the liprin-α/LAR-RPTP interaction.

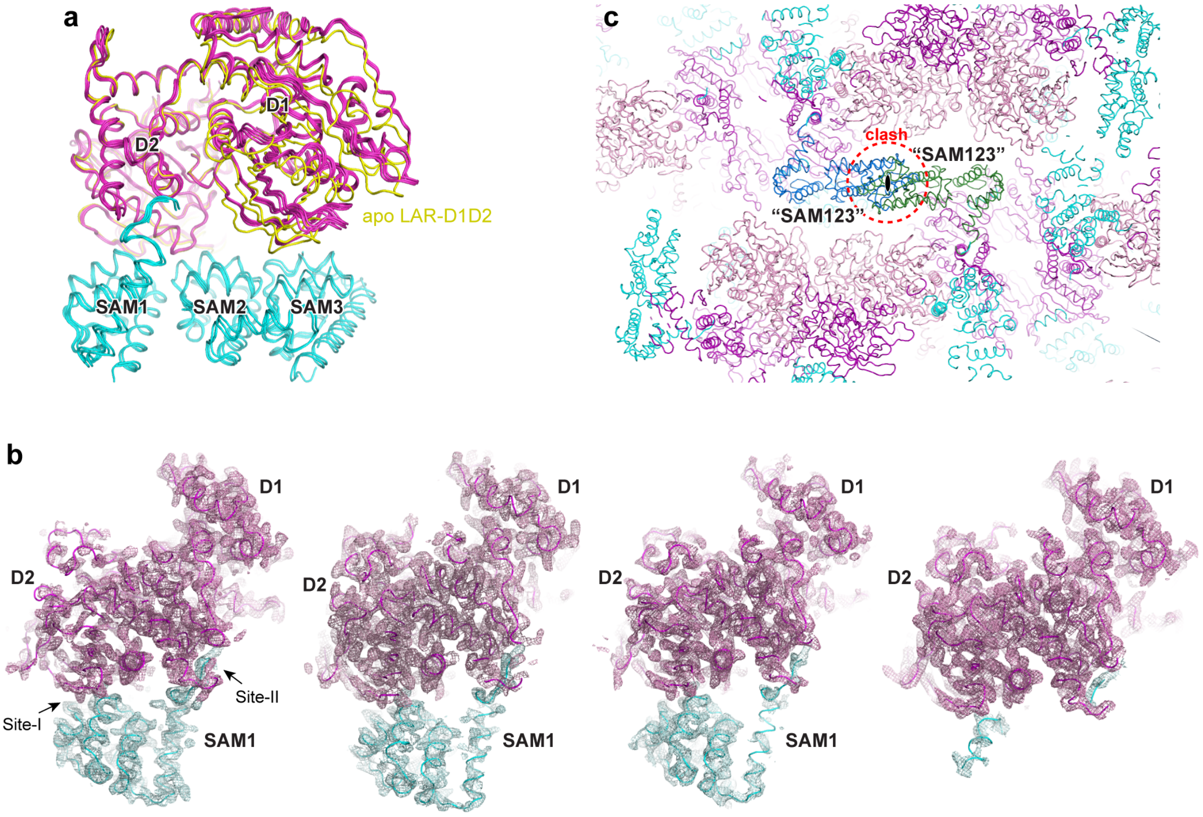

Unmodeled regions

LAR D1D2	Chain D:	1648-1655 & 1906-1907	Chain A:	1651-1653 & 1906-1907	Chain B:	1651-1655 & 1904-1907	Chain C:	1653-1654 & 1905-1907
Liprin-α3 SAM123	Chain H:	906-950, 1029-1036, 1052-1059, 1088-1098, & 1109-1126	Chain E:	906-950 & 1117-1126	Chain F:	806, 907-950 & 1115-1126	Chain G:	806, 810-852 & 865-1126

Supplementary Figure 4. Structural analysis of the liprin-α3_SAM123/LAR_D1D2 complex in crystal. (**a**) Structural comparison of the LAR_D1D2 structure in the apo form and the α3_SAM123 bound form. Four complex structures in one asymmetric unit are superimposed. (**b**) Composite-omit maps of the four α3_SAM123 and LAR_D1D2 complex structures in one asymmetric unit. The $2F_o$-F_c densities are contoured in 1.0σ with the structures superimposed. Corresponding unmodeled regions were listed to the above complex structures. Notably, one α3_SAM123 chain (chain G) is mostly unmodeled. (**c**) Crystal packing analysis. The chain G in crystal was replaced by an intact α3_SAM123 molecule (blue) to form a complex with LAR_D1D2 (chain C). The new α3_SAM123 molecule clashes into its symmetric molecule (green). Therefore, the fourth SAM123 molecule does not fit in the asymmetric unit in a fixed orientation. The crystallographic two-fold axis was indicated by an ellipse.

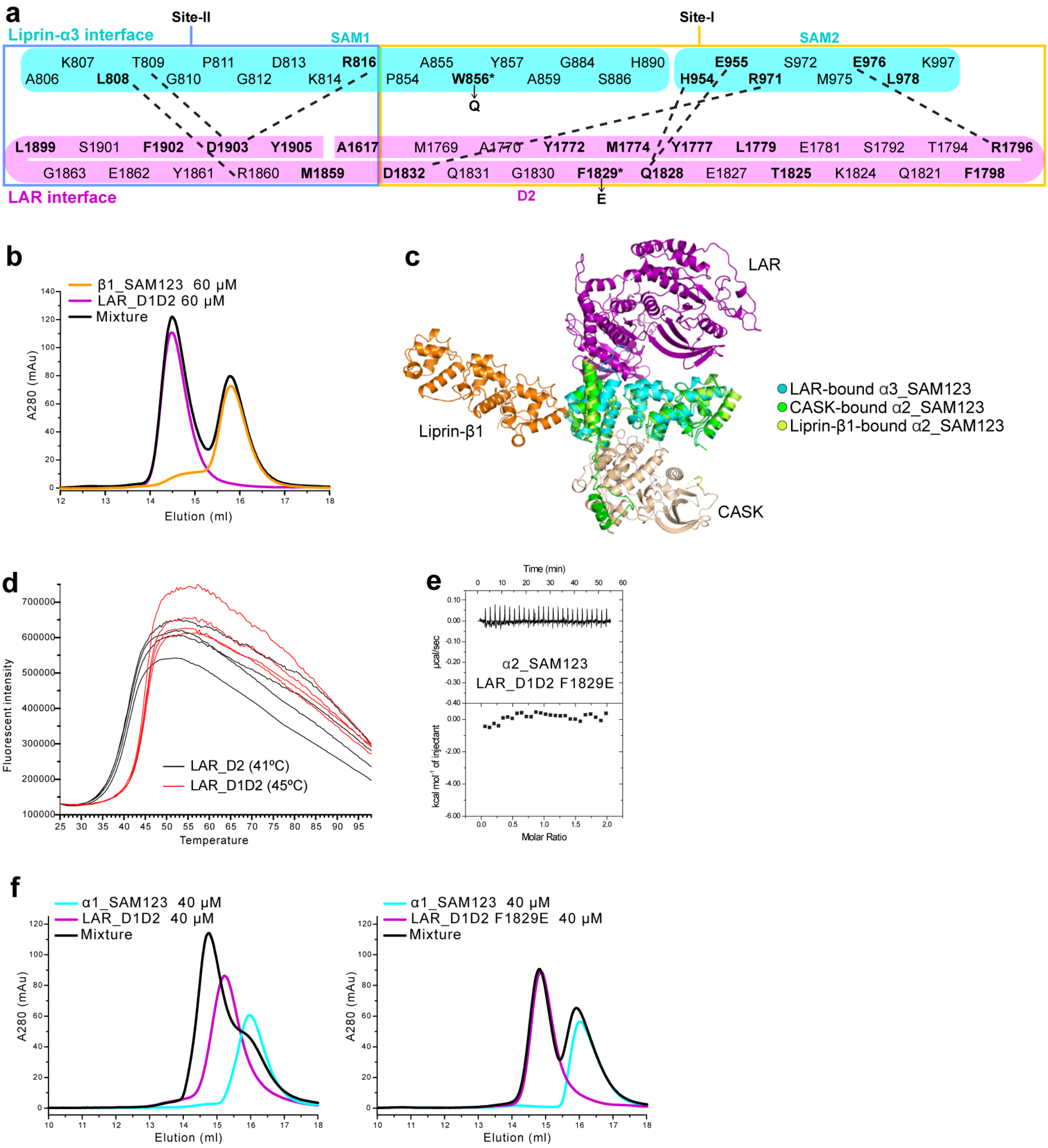

Supplementary Figure 5. Interaction analysis between LAR and different liprin proteins. (**a**) The list of residues locating at the interfaces in liprin-α3 and LAR. The interface residues shown in Fig. 2c,d were highlighted with bold fonts. The two key interface residues (W856 in liprin-α3 and F1829 in LAR) were mutated in this study were marked with asterisks. (**b**) Analytical gel filtration analysis showing no detectable binding of LAR to liprin-β. (**c**) Structural comparison of liprin-α_SAM123 structures in complex with three binding partners respectively. (**d**) Thermal stability analysis of LAR_D2 and LAR_D1D2 by using ThermoFluor assay. (**e**) ITC-based analysis of the binding of α2_SAM123 to the F1829E mutant of LAR_D1D2. (**f**) Analytical gel filtration analysis of the interactions between α1_SAM123 and LAR_D1D2 or its F1829E mutant.

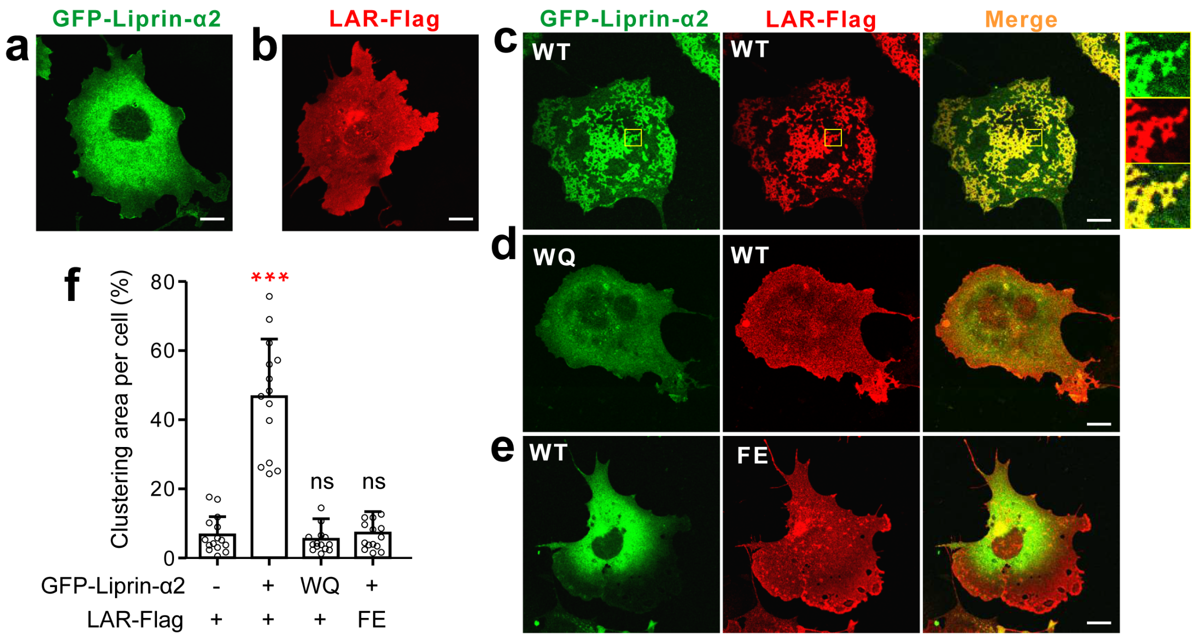

Supplementary Figure 6. Liprin-α2 promotes the clustering of LAR. (**a**, **b**) Cell imaging of over-expressed GFP-liprin-α2 (**a**) and LAR-Flag (**b**) in COS7 cells. Scale bar, 10 µm. (**c**-**e**) Cells co-transfected with GFP-tagged liprin-α2 or its mutant and Flag-tagged LAR or its mutant. "WQ" and "FE" indicate the W916Q mutant of liprin-α2 and the F1829E mutant of LAR, respectively. Regions of interesting were highlighted by yellow boxes, enlarged 4 times, and aligned on the right of merged image. Scale bar, 10 µm. (**f**) Quantification of the LAR clustering levels as shown in **a**-**e**. ~15 cells per experimental condition.

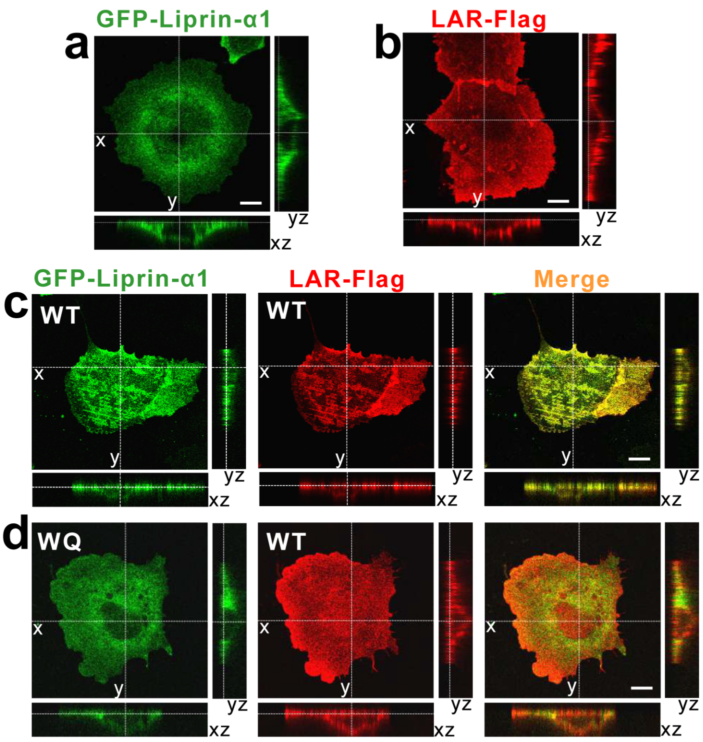

Supplementary Figure 7. Confocal xz & yz sectioning microscopic analysis. (**a**, **b**) Over-expressed liprin-α1 and LAR in COS7 cells. Reconstruction (z-axis) from confocal sections of COS7 cells transfected with GFP-liprin-α1 (**a**) and LAR-Flag (**b**). (**c**, **d**) Reconstruction (z-axis) from confocal sections of COS7 cells co-transfected with GFP-liprin-α1 or its mutant and LAR-Flag. "WQ" indicate the W896Q mutant of liprin-α1. Scale bar, 10 μm.

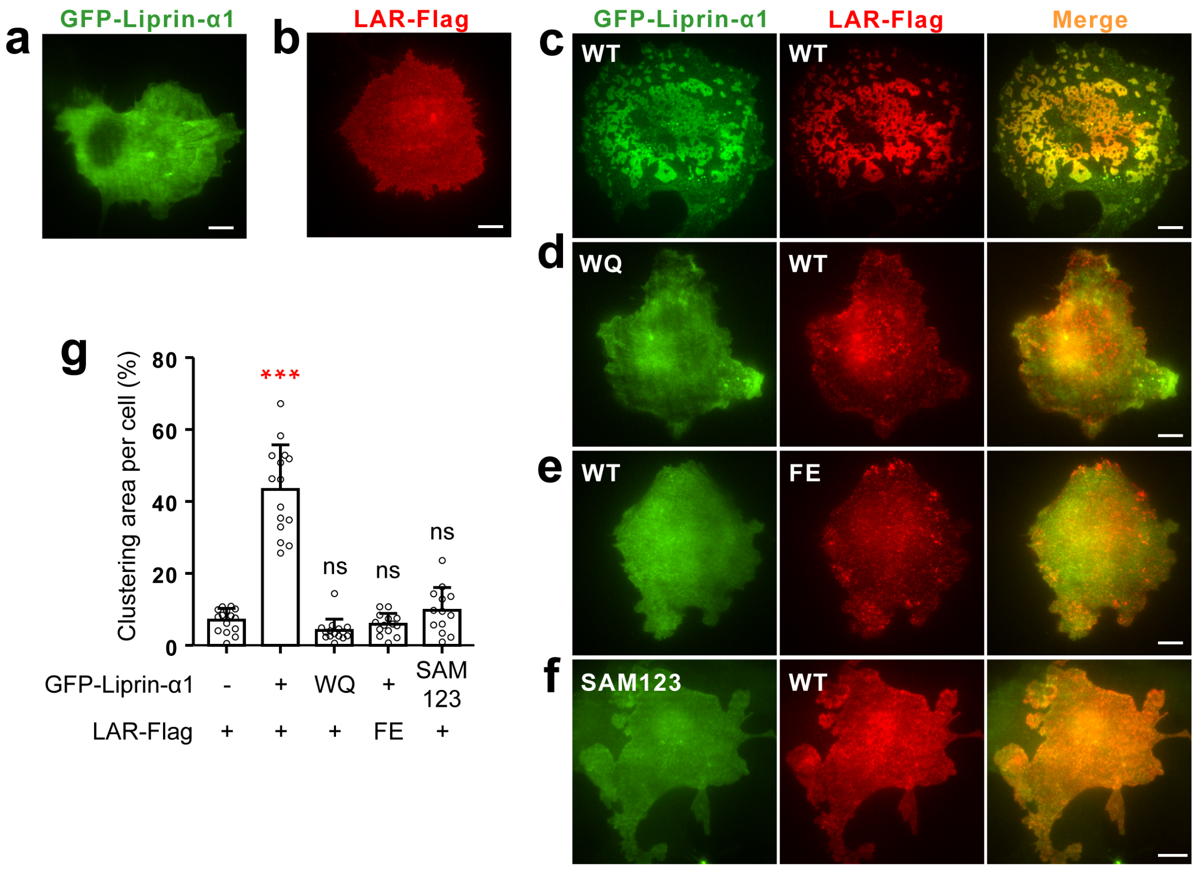

Supplementary Figure 8. TIRF imaging analysis of the LAR clustering. (**a, b**) COS7 cells overexpressing GFP-liprin-α1 (**a**) and LAR-Flag (**b**). (**c-f**) COS7 cells co-transfected with GFP-liprin-α1 or its variants and LAR-Flag or its variants. "WQ" and "FE" indicate the W896Q mutant of liprin-α1 and the F1829E mutant of LAR, respectively. Scale bar, 10 µm. (**g**) Quantification of the LAR clustering levels as shown in **a-f**. ~15 cells per experimental condition.

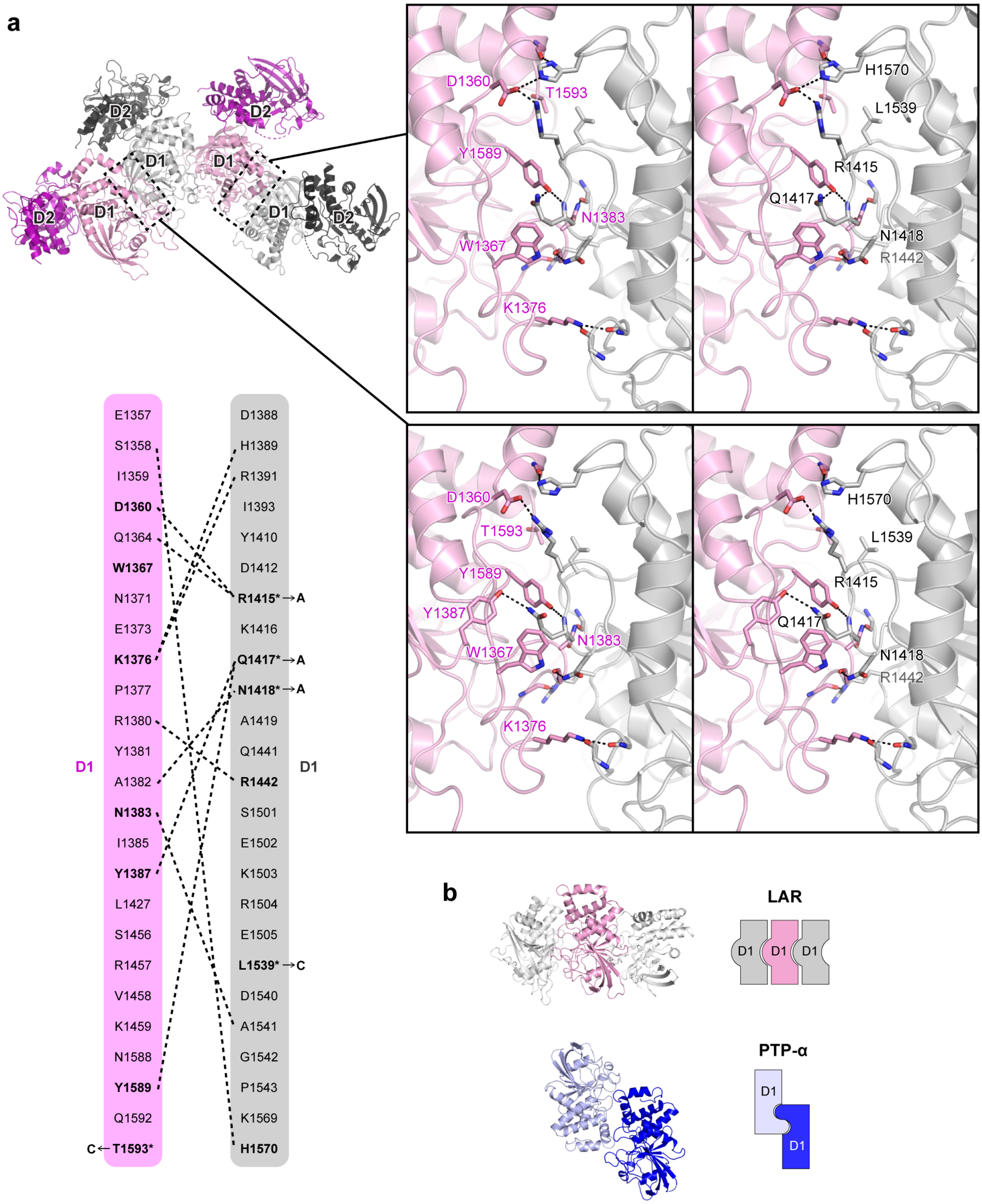

Supplementary Figure 9. The D1/D1 packings. (**a**) The D1/D1 packings found in the four LAR-D1D2 protomers in one asymmetric unit. Two D1/D1 packing interfaces were zoomed in and shown as stereoview views. The complete interface residues were shown. Hydrogen bonds and salt bridges are indicated by dashed lines. Resides involved in our mutagenesis study were indicated by asterisks. (**b**) Structural comparison of two types of the D1/D1 packings

found in LAR and another RPTP, PTP-α (PDB ID: 1YFO), which forms a homodimer through an N-terminal "wedge"[53].

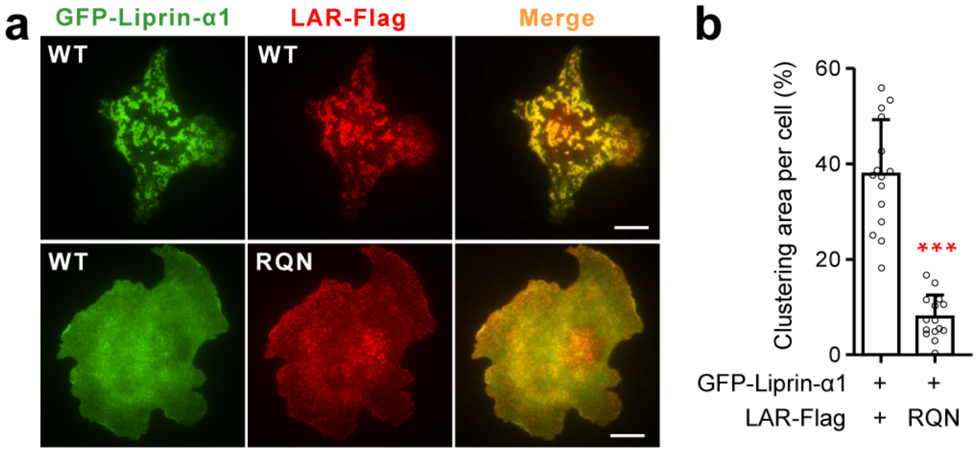

Supplementary Figure 10. TIRF imaging analysis showing the D1/D1 packing-mediated clustering of LAR. (**a**) COS7 cells were co-transfected with GFP-liprin-α1 and LAR-Flag or its RQN mutant (R1415A/Q1417A/N1418A). Scale bar, 10 μm. (**b**) Quantification of the LAR clustering levels as shown in **a**. ~15 cells per experimental condition.

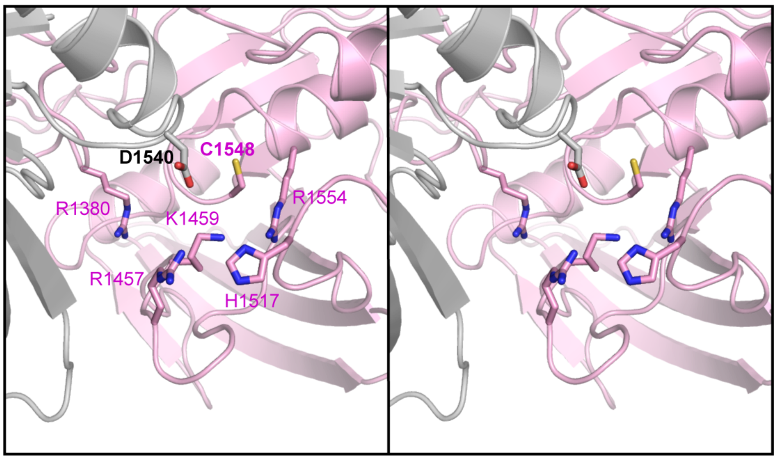

Supplementary Figure 11. Molecular details of the substrate-binding pocket in the D1 domain with the D1/D1 packing. The $\alpha3_{D1}/\beta11_{D1}$-loop close to the D1/D1 interface was shown to block the entry of substrates to the pocket.

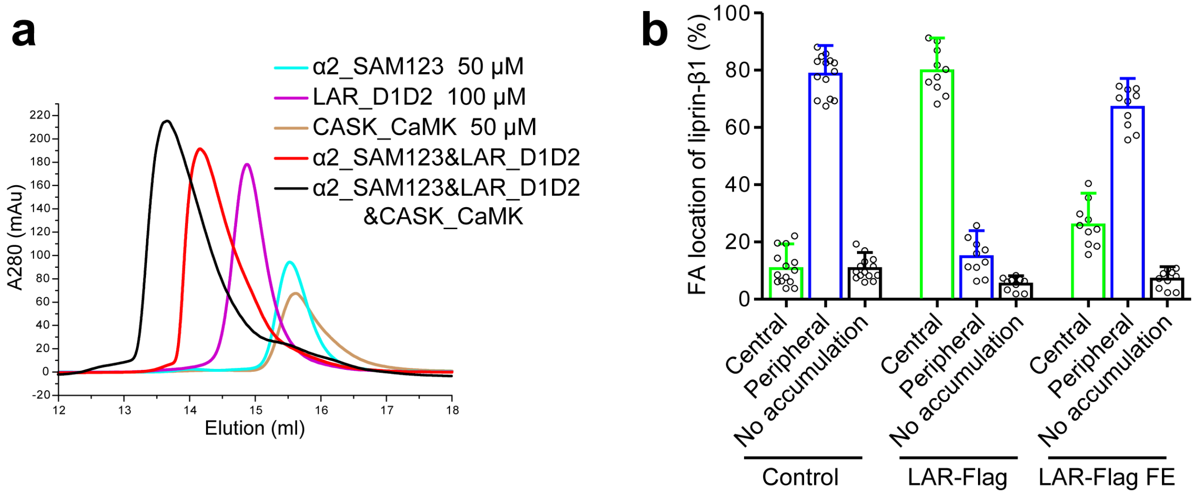

Supplementary Figure 12. The SAM123-mediated interactions. (**a**) Analytical gel filtration analysis showing a tripartite complex formation of liprin-α2, LAR, and CASK. (**b**) Quantitative analysis of FA location of liprin-β1 per cell as shown in Fig. 6f-h. The FA locations of liprin-β1 were classified into three types: the peripheral regions of the FA, the central regions of the FA and no accumulation of liprin-β1 at the FA. The FA number in each type was counted and then divided by total FA number per cell. 10-14 cells per experimental condition.

Supplementary Table 1. Summary of protein constructs used in this study

Protein	Fragment	Mutant	Vector	Name in this paper
Human LAR	1-1907		pCDNA3.1	LAR
	1-1907	F1829E	pCDNA3.1	LAR FE
	1-1907	R1415A, Q1417A, N1418A	pCDNA3.1	LAR RQN
	1332-1907		pCDNA3.1	LAR_D1D2
	1332-1907		pET.SUMO	LAR_D1D2
	1332-1907	F1829E	pET.SUMO	LAR_D1D2 FE
	1332-1907	F1829E	pET.SUMO	LAR_D1D2 FE
	1332-1907	C1548S	pET.SUMO	LAR_D1D2 CS
	1332-1907	L1539C	pET.SUMO	LAR_D1D2 L1539C
	1332-1907	T1593C	pET.SUMO	LAR_D1D2 T1593C
	1332-1907	L1593C, T1593C	pET.SUMO	LAR_D1D2 L1539C/T1593C
	1602-1898		pET.SUMO	LAR_D2
Human PTP-σ	1355–1930		pET.32M.3C	PTP-σ_D1D2
Human PTP-δ	1339-1912		pET.32M.3C	PTP-δ_D1D2
Human liprin-α1	1-1202		pEGFPC1	liprin-α1
	1-1202	W896Q	pEGFPC1	liprin-α1 WQ
	847-1136		pEGFPC1	liprin-α1_SAM123
	847-1136		pET.32M.3C	liprin-α1_SAM123
Human liprin-α2	1-1257		pEGFPC1	liprin-α2
	1-1257	W916Q	pEGFPC1	liprin-α2 WQ
	866-1193		pET.32M.3C	liprin-α2_SAM123
	866-1001		pET.32M.3C	liprin-α2_SAM1
	1002-1193		pET.32M.3C	liprin-α2_SAM23
	866-1193 (Δ976-1012)		pET.32M.3C	liprin-α2_SAM123 ΔInsertion
	879-1193		pET.32M.3C	liprin-α2_SAM123 ΔN
Mouse liprin-α3	806-1126		pET.32M.3C	liprin-α3_SAM123
	806-1126	W856Q	pET.32M.3C	liprin-α3_SAM123 WQ
Mouse liprin-α4	797-1117		pET.32M.3C	liprin-α4_SAM123
Mouse liprin-β1	593-853		pET.32M.3C	liprin-β1_SAM123
Mouse CASK	1-345		pET.32M.3C	CASK_CaMK
Human paxillin	1-557		pEGFPC1	paxillin

Supplementary Table 2. Summary of primers used in this study

Primer Name	Sequence
pcDNA3.1_lar_1_f	TTAAACTTAAGCTTGGTACCATGGCCCCTGAGCCAGCC
pcDNA3.1_lar_1907_r	AGTCCATGGTGGCGGATCCCGTTGCATAGTGGTCAAAG
pcDNA3.1_lar_1332_f	TTAAACTTAAGCTTGGTACCATCCCCATCACCGACCTG
pSUMO_lar_1332_f	ATGGATCCATCCCCATCACCGACCTG
pSUMO_lar_1907_r	GAGTGCGGCCGCTACGTTGCATAGTGGTCAAAG
pSUMO_lar_1602_f	ATGGATCCGAGGCGCTGCTGGAGGCTG
pSUMO_lar_1898_r	GAGTGCGGCCGCTAGTACTCCAGGGCCGCACGG
PTP-σ_1335_f	ATGGATCCCTCAGGAGCCCCCTCAGGG
PTP-σ_1930_r	GAGTGCGGCCGCTAGGTTGCATAGTGGTCAAAG
PTPD_1339_f	CTGTTCCAGGGGCCCAGATCTATCTTGGAACTTGCAGAC
PTPD_1912_r	TGCTCGAGTGCGGCCGCAAGCTTACGTTGCATAGTGGTC
Liprin-α1_1_f	CTGAATTCATGATGTGCGAGGTGATG
Liprin-α1_1202_r	CTCTCGAGTTAGCAGGAGTAAGTCCTG
Liprin-α1_1046_r	CTACTCGAGCTATTTTATTTCACTCTGACTTTC
Liprin-α1_847_f	GTGAATTCAAATTGGGGGGACAGGCTG
Liprin-α1_1136_r	TGCTCGAGTTATCTCCTAAAGCTTTTATC
Liprin-α2_1_f	CAGAATTCATGATGTGTGAAGTGATGCCC
Liprin-α2_1257_r	CTCTCGAGTCAACATGAGTATGTGCG
Liprin-α2_866_f	CAGAATTCGGCAAACTCGGAACTCAAG
Liprin-α2_1193_r	TGCTCGAGTCAACGTCTGAAGTTCTTGTCATC
Liprin-α2_1001_r	TGCTCGAGTCATTCCTCAGATTCTTTCG
Liprin-α2_1002_f	CAGAATTCGGAAGCTGGGCCCAGTGTC
Liprin-α2_(Δ976-1012)_f	CCTCCAACATCTCGAACTACCCTGGCTTATGGAGATATG
Liprin-α2_(Δ976-1012)_r	CATATCTCCATAAGCCAGGGTAGTTCGAGATGTTGGAGG
Liprin-α2_879_f	CAGAATTCAAGAAAAAGCATGAACTTC
Liprin-α3_806_f	GAGGATCCGCCAAGCTGACTGGTCCAGGA
Liprin-α3_1126_r	CGAAGCTTAGCGGCTGAAGGACTTGGCAC
Liprin-α4_797_f	GAGGATCCAAGTTGGGGACCCAGGCAGAG
Liprin-α4_1117_r	CTCTCGAGTTATCGGCGGAACACCTTCTCTTC
Liprin-β1_593_f	GTGGATCCGCAGGACCCAGGCTTGGTTG
Liprin-β1_853_r	TAGTCGACTTATCGCTTCTGGTGCTGGGCC
CASK_1_f	CAGAATTCATGGCCGACGACGACGTGC
CASK_345_r	TGCTCGAGTCATCTTTCTGCTGCTAGAAG
paxillin_1_f	CAGAATTCATGGACGACCTCGACGCCCTGC
paxillin_557_r	TGCTCGAGCTAGCAGAAGAGCTTGAGGAAGC
