# Supplementary material for: Structural basis of liprin-α-promoted LAR-RPTP clustering for modulation of phosphatase activity
Source: Nat Commun. 2020 Jan 10;11:169. doi: 10.1038/s41467-019-13949-x (PMC6954185; doi:10.1038/s41467-019-13949-x)
Supplement: Supplementary file 3 — Reporting Summary [file 41467_2019_13949_MOESM3_ESM.pdf]

nature research

Corresponding author(s): Zhiyi Wei

Last updated by author(s): Nov 24, 2019

Reporting Summary

Nature Research wishes to improve the reproducibility of the work that we publish. This form provides structure for consistency and transparency in reporting. For further information on Nature Research policies, see Authors & Referees and the Editorial Policy Checklist .

Statistics

For all statistical analyses, confirm that the following items are present in the figure legend, table legend, main text, or Methods section.

n/a	Confirmed	
☐	☒	The exact sample size (*n*) for each experimental group/condition, given as a discrete number and unit of measurement
☐	☒	A statement on whether measurements were taken from distinct samples or whether the same sample was measured repeatedly
☐	☒	The statistical test(s) used AND whether they are one- or two-sided<br>*Only common tests should be described solely by name; describe more complex techniques in the Methods section.*
☒	☐	A description of all covariates tested
☒	☐	A description of any assumptions or corrections, such as tests of normality and adjustment for multiple comparisons
☐	☒	A full description of the statistical parameters including central tendency (e.g. means) or other basic estimates (e.g. regression coefficient) AND variation (e.g. standard deviation) or associated estimates of uncertainty (e.g. confidence intervals)
☒	☐	For null hypothesis testing, the test statistic (e.g. *F*, *t*, *r*) with confidence intervals, effect sizes, degrees of freedom and *P* value noted<br>*Give P values as exact values whenever suitable.*
☒	☐	For Bayesian analysis, information on the choice of priors and Markov chain Monte Carlo settings
☒	☐	For hierarchical and complex designs, identification of the appropriate level for tests and full reporting of outcomes
☒	☐	Estimates of effect sizes (e.g. Cohen's *d*, Pearson's *r*), indicating how they were calculated

Our web collection on statistics for biologists contains articles on many of the points above.

Software and code

Policy information about availability of computer code

Data collection	X-ray diffraction data were processed and scaled using HKL3000 software.
Data analysis	The initial phase of the crystal structure was determined by molecular replacement in PHASER. The model was refined in PHENIX-1.9. COOT-0.8 was used for model rebuilding and adjustments. The model quality was check by MolProbity. All structure figures were prepared by using PyMOL-2.0.

For manuscripts utilizing custom algorithms or software that are central to the research but not yet described in published literature, software must be made available to editors/reviewers. We strongly encourage code deposition in a community repository (e.g. GitHub). See the Nature Research guidelines for submitting code & software for further information.

Data

Policy information about availability of data

All manuscripts must include a data availability statement. This statement should provide the following information, where applicable:

- Accession codes, unique identifiers, or web links for publicly available datasets
- A list of figures that have associated raw data
- A description of any restrictions on data availability

The coordinates and structure factors for the liprin-3_SAM123/LAR_D1D2 complex have been deposited in the Protein Data Bank (PDB) under the accession codes 6KR4. All other data supporting the findings of this study are available within the paper and its supplementary information files.

Field-specific reporting

Please select the one below that is the best fit for your research. If you are not sure, read the appropriate sections before making your selection.

☒

Life sciences study design

All studies must disclose on these points even when the disclosure is negative.

Sample size	No sample size was predetermined.
Data exclusions	A few data point in ITC titration experiments were excluded from curve fitting due to abnormally heat changes.
Replication	Experiments were replicated successfully.
Randomization	N/A
Blinding	No blinding was performed in this study.

Reporting for specific materials, systems and methods

We require information from authors about some types of materials, experimental systems and methods used in many studies. Here, indicate whether each material, system or method listed is relevant to your study. If you are not sure if a list item applies to your research, read the appropriate section before selecting a response.

Materials & experimental systems

n/a	Involved in the study	
☐	☒	Antibodies
☐	☒	Eukaryotic cell lines
☒	☐	Palaeontology
☒	☐	Animals and other organisms
☒	☐	Human research participants
☒	☐	Clinical data

Methods

n/a	Involved in the study	
☒	☐	ChIP-seq
☒	☐	Flow cytometry
☒	☐	MRI-based neuroimaging

Antibodies

Antibodies used	For immuno-fluorescence, primary antibodies against Flag (Sigma, F1804), liprin-α1 (ProteinTech, 14175-1-AP and Santa cruz, SC376141), liprin-β1 (ATLAS, HPA001924), LAR (Sigma, GW21469), and pTyr(PY99) (Santa cruz, SC7020) were used. Dye-conjugated secondary antibodies against Rabbit IgG (H+L) (Thermo, A-11012), Mouse IgG (H+L) (Thermo, A-21203&A-21046) and Chicken IgY (H+L) (Thermo, A-21203, A-21449) were used.
Validation	The validation statement of each primary antibody can be found on the manufacturer's website.

Eukaryotic cell lines

Policy information about cell lines

Cell line source(s)	293T and COS-7 cells were obtained from ATCC.
Authentication	Microscopes were used to distinguished cell lines based on their morphology.
Mycoplasma contamination	No mycoplasma contamination was found.
Commonly misidentified lines (See ICLAC register)	N/A
